# The Role of Gut Microbiota-Derived Lithocholic Acid, Deoxycholic Acid and Their Derivatives on the Function and Differentiation of Immune Cells

**DOI:** 10.3390/microorganisms11112730

**Published:** 2023-11-08

**Authors:** Yoshimitsu Kiriyama, Hiromi Nochi

**Affiliations:** 1 Kagawa School of Pharmaceutical Sciences, Tokushima Bunri University, Sanuki 769-2193, Japan; nochi@kph.bunri-u.ac.jp; 2 Institute of Neuroscience, Tokushima Bunri University, Sanuki 769-2193, Japan

**Keywords:** bile acids, DCA, LCA, isoalloLCA, isoDCA, FXR, RORγt, NR4A1, FOXP3, TGR5, Th1, Th17, Treg

## Abstract

A wide variety and large number of bacterial species live in the gut, forming the gut microbiota. Gut microbiota not only coexist harmoniously with their hosts, but they also induce significant effects on each other. The composition of the gut microbiota can be changed due to environmental factors such as diet and antibiotic intake. In contrast, alterations in the composition of the gut microbiota have been reported in a variety of diseases, including intestinal, allergic, and autoimmune diseases and cancer. The gut microbiota metabolize exogenous dietary components ingested from outside the body to produce short-chain fatty acids (SCFAs) and amino acid metabolites. Unlike SCFAs and amino acid metabolites, the source of bile acids (BAs) produced by the gut microbiota is endogenous BAs from the liver. The gut microbiota metabolize BAs to generate secondary bile acids, such as lithocholic acid (LCA), deoxycholic acid (DCA), and their derivatives, which have recently been shown to play important roles in immune cells. This review focuses on current knowledge of the role of LCA, DCA, and their derivatives on immune cells.

## 1. Introduction

The mucous membranes of the oral cavity, stomach, gut, and skin form the boundary between the inside and outside of the human body. These mucosal and skin surfaces are inhabited by numerous bacteria [1,2]. In particular, a wide variety and large number of bacterial species live in the gut and form the gut microbiota. The estimated number of bacterial cells in the human body is approximately 3.8 × 10^13^ [1]. Furthermore, the estimated number of human cells is approximately 3 × 10^13^. Thus, the number of bacteria in the human body is equivalent to the number of human cells. In addition, the total mass of bacteria in the human body is estimated to be approximately 0.2 kg [1]. Although a wide variety of bacteria are present in the human gut microbiota, the predominant bacteria are Bacteroidetes, Firmicutes, Actinomycetes, Proteobacteria, and Velcomicrobia at the phylum level. Moreover, the environments of the small and large intestines are significantly different, with varying types of intestinal bacteria inhabiting each part. More specifically, the predominant bacterial families in the small intestine are Lactobacillaceae and Enterobacteriaceae, while those in the colon are Bacteroidaceae, Prevotellaceae, Rikenellaceae, Lachnospiraceae, and Ruminococcaceae [3]. Human gut microbiota and humans not only coexist, but they also influence each other. The composition of the gut microbiota in humans can be affected by environmental factors such as diet and antibiotic intake. The development of metagenomic analysis of the gut microbiota has allowed researchers to perform comparative analyses of the gut microbiota between healthy and diseased individuals, who have in turn identified a correlation between various diseases and changes in the gut microbiota composition. For instance, alterations in the composition of the gut microbiota have been reported in a variety of diseases, including intestinal diseases (e.g., inflammatory bowel disease and colorectal cancer), liver diseases (e.g., nonalcoholic fatty liver disease and liver cancer), systemic metabolic diseases (e.g., diabetes and atherosclerosis), immune-related diseases (e.g., allergic and autoimmune diseases), and neuropsychiatric diseases (e.g., Alzheimer’s and depression) [4,5,6]. In addition, changes in the composition of gut microbiota have been associated with the persistence of symptoms in many patients with post-coronavirus disease 2019 (COVID-19) symptoms, also known as acute post-COVID-19 syndrome (PACS) or long-term COVID [7,8,9]. 

However, various metabolites produced by the activity of the gut microbiota have also been found to affect their respective hosts. The gut microbiota metabolize exogenous dietary components ingested from outside the body to produce short-chain fatty acids (SCFAs), such as propionic acid, butyric acid, and acetic acid, and amino acid metabolites, such as serotonin (5-HT), dopamine, histamine, and gamma-amino butyric acid (GABA). These SCFAs have a variety of important physiological and pathological roles [10,11]. In addition, 5-HT, dopamine, histamine, and GABA, which are involved in neurotransmission, are produced by the gut microbiota as amino acid metabolites [12,13]. 

**Figure 1:**
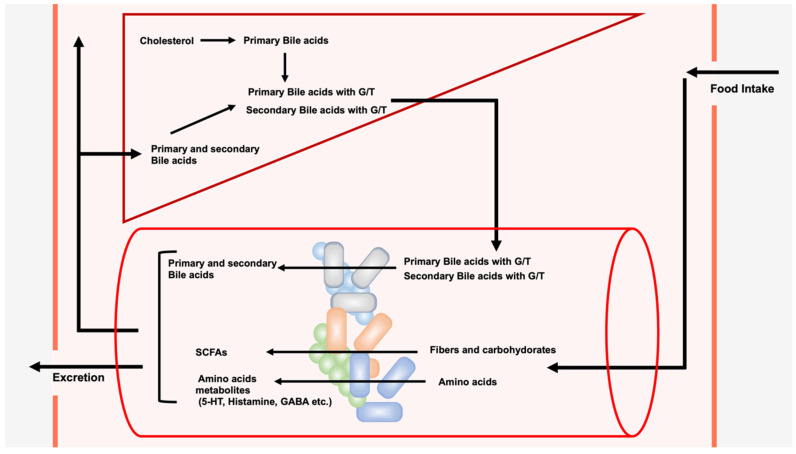
Modification of exogenous dietary components and endogenous bile acids by the gut microbiota. The gut microbiota metabolize exogenous dietary components ingested from outside the body to produce short-chain fatty acids (SCFAs), such as propionic acid, butyric acid, and acetic acid, and amino acid metabolites, such as serotonin (5-HT), dopamine, histamine, and gamma-amino butyric acid (GABA). The gut microbiota also metabolize endogenous bile acids (BAs). Cholesterol is converted to cholic acid (CA) or chenodeoxycholic acid (CDCA) in the liver. These newly produced BAs in the liver are called primary BAs, which are then conjugated with glycine or taurine (G/T). Conjugated primary BAs are secreted into the intestine upon food intake, and they are subsequently deconjugated and then converted to secondary BAs. Approximately 95% of BA in the intestine is reabsorbed and transported to the liver, while the remaining BA is excreted in the feces. A portion of BA reabsorbed from the intestine is transferred to the systemic circulation.

Unlike SCFAs and amino acid metabolites stemming from diets, bile acids (BAs) are synthesized from cholesterol in the liver (Figure 1). The newly produced BAs in the liver are called primary BAs, and chenodeoxycholic acid (CDCA) and cholic acid (CA) are the major primary BAs in humans [14,15]. In addition to these two BAs, previous research has shown that muricholic acids and hyocholic acids are produced in mice [15,16] and pigs [17], respectively. The gut microbiota metabolize these primary BAs to produce secondary bile acids, such as lithocholic acid (LCA), deoxycholic acid (DCA), and ursodeoxycholic acid (UDCA) [18,19,20], which play important roles in mitochondria and autophagy at the cellular level [21,22]. Furthermore, they are physiologically and pathologically important to the host as well as to the composition of the gut microbiota [14,23]. In particular, among secondary BAs, LCA, DCA, and their derivatives have recently been shown to have a significant effect on immune cells [24,25,26,27,28,29,30]. This review focuses on current knowledge of the role of LCA, DCA, and their derivatives on immune cells.

## 2. LCA, DCA, and Their Derivatives from Gut Microbiota

**Figure 2:**
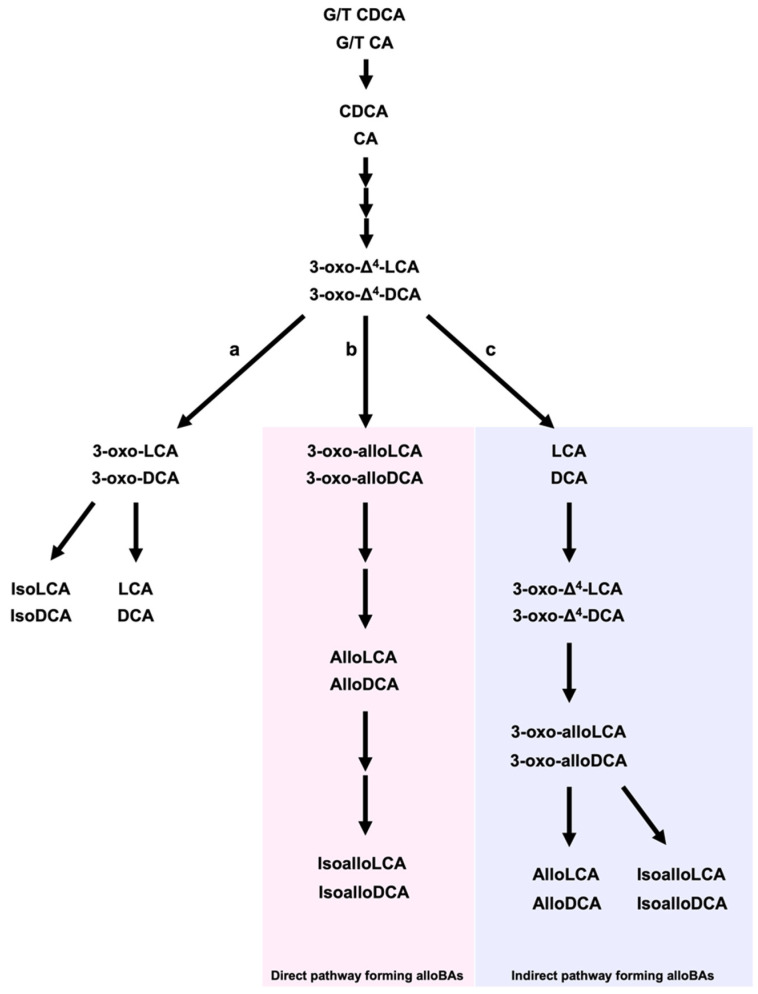
The metabolism of bile acids by gut microbiota to produce LCA, DCA, and their derivatives. In the intestine, glycine or taurine-conjugated primary BAs (CDCA and CA) are deconjugated by gut bacteria. CDCA and CA are then converted to 3-oxo-Δ^4^-LCA and 3-oxo-Δ^4^-DCA, respectively. (a) 3-oxo-Δ^4^-LCA and 3-oxo-Δ^4^-DCA are converted to 3-oxoLCA and 3-oxoDCA, respectively, which are then converted to LCA and DCA, respectively. There are direct (b) and indirect (c) pathways to produce alloBAs. The direct pathway is conducted by a single bacterial strain, while the indirect pathway is carried out by multiple bacterial strains. In the direct pathway (b), 3-oxo-Δ^4^-LCA and 3-oxo-Δ^4^-DCA are converted to alloLCA and alloDCA, respectively, which are in turn converted to isoalloLCA and isoalloDCA, respectively. In the indirect pathway (c), 3-oxo-Δ^4^-LCA and 3-oxo-Δ^4^-DCA are converted to LCA and DCA, and then to 3-oxo-Δ^4^-LCA and 3-oxo-Δ^4^-DCA, respectively. 3-oxo-Δ^4^-LCA and 3-oxo-Δ^4^-DCA are then converted to alloBAs.

Primary BAs, such as CDCA and CA, are primarily synthesized in the liver from cholesterol via two synthetic pathways, namely the classical (or neutral) pathway and the alternative (or acidic) pathway [14,31]. More than 16 enzymes, including cytochrome P450 7A1 (CYP7A1), CYP8B1, and CYP27A1, are involved in primary BA synthesis in the liver [32,33]. Primary BAs are then conjugated with glycine or taurine by bile acid-CoA amino acid N-acyltransferase in the liver [34], and they are transferred to hepatic bile canaliculi via the bile salt export pump or multidrug resistance-associated protein 2. Consequently, BAs in the gallbladder are secreted into the small intestine in response to food intake [35,36], whereas glycine or taurine-conjugated BAs are deconjugated by bile salt hydrolases (BSHs) in gut bacteria (Figure 2) [37]. Various gut bacteria in the ileum and colon, including *Lactobacillus*, *Bifidobacterium*, *Enterococcus*, *Clostridium*, and *Bacteroides* spp., have been shown to possess BSH enzyme activity [38,39,40,41,42,43]. *Lachnoclostridium scindens* (formerly *Clostridium scindens*), *Peptacetobacter hiranonis* (formerly *Clostridium hiranonis*), and *Lachnoclostridum hylemonae* (formerly *Clostridium hylemonae*) can 7α-dehydroxylation of BAs. 7α-dehydroxylation of the CDCA and CA is carried away by enzymes encoded in the bile acid-inducible (*bai*) operon to produce 3-oxo-Δ^4^-LCA and 3-oxo-Δ^4^-DCA, respectively [19,20,44]. Both 3-xo-Δ^4^-LCA and 3-oxo-Δ^4^-DCA are intracellular intermediates that are not released in appreciable amounts into the environment.

**Figure 3:**
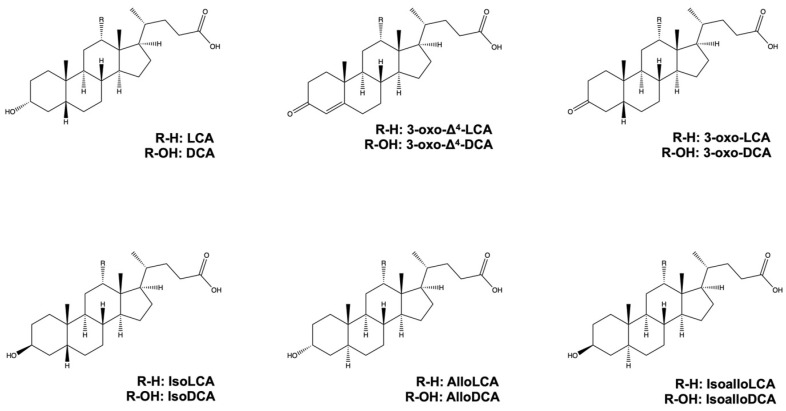
Chemical structures of LCA, DCA, and their derivatives.

There are both direct and indirect pathways to produce alloBAs, which are essentially BAs with a flat shape due to the trans-orientation of the A and B rings of BAs [45]. On the one hand, the direct pathway is conducted by a single bacterial strain, while on the other hand, the indirect pathway is carried out by multiple bacterial strains [19,45]. In the direct pathway, 5ɑ-reductase and BaiA1 (3ɑ-hydroxy bile acid-CoA-ester 3-dehydrogenase 1) convert 3-oxo-Δ^4^-LCA and 3-oxo-Δ^4^-DCA to alloLCA and alloDCA, respectively. AlloDCA is subsequently converted to isoalloDCA by 3α-HSDH and 3β-HSDH in *Lachnoclostridium scindens.* In addition, isoalloLCA can be indirectly produced from 3-oxo-LCA by eleven bacterial genera, namely *Bacillus*, *Bacteroides*, *Bifidobacterium*, *Catenibacterium*, *Collinsella*, *Eggerthella*, *Lachnospira*, *Lactobacillus*, *Parabacteroides*, *Peptoniphilus*, and *Mediterraneibacter* [29]. Moreover, a mixture of *Lachnoclostridium scindens*, which can produce LCA from CDCA [46], *Eggerthella lenta*, which can produce 3-oxo-LCA from LCA [47], and *Parabacteroides merdae*, which can produce isoalloLCA from 3-oxo-LCA [29] can synthesize isoalloLCA from LCA. In addition, 5β-reductase, 5α-reductase, and 3β-HSDH enzymes can synthesize isoalloLCA from 3-oxo-LCA, while 3-oxo-Δ^4^-LCA can be produced from 3-oxo-LCA by 5β-reductase. 3-oxo-alloLCA is then produced from 3-oxo-Δ^4^-LCA by 5α-reductase. IsoalloLCA is produced from 3-oxo-alloLCA by 3β-HSDH [29,48]. 5α-reductase converts 3-oxo-Δ^4^-DCA to 3-oxo-DCA and 5β-reductase converts 3-oxo-Δ^4^-DCA to 3-oxo-alloDCA. 3α-HSDH converts 3-oxo-DCA and 3-oxo-alloDCA to DCA and alloDCA, respectively [19,45]. IsoDCA and isoLCA are generated from DCA and LCA by *3β-HSDH*, respectively [20]. The chemical structures of LCA, DCA, and their derivatives are shown in Figure 3.

Approximately 95% of BA in the intestine is reabsorbed and transported to the liver, and the remainder is excreted in the feces. LCA can be sulfonated and converted to LCA 3-sulfate (LCA-3-S) by sulfotransferase in the liver [49,50]. 

## 3. Role of LCA, DCA, and Their Derivatives on Immune cells

### 3.1. Antigen-Presenting Cells and Helper T Cell Subset

A cluster of differentiation (cd) molecules are extracellular proteins of T cells. T cells are broadly classified into helper T (Th) cells, which express CD4, and cytotoxic T lymphocytes (CTLs), which express CD8. T cells that have just emerged from the thymus to peripheral tissues that have not yet encountered antigens are called naïve T cells. The differentiation of naïve Th CD4^+^ cells into various Th cell subsets begins when naïve CD4^+^ Th cells encounter antigen-presenting cells (APCs), such as dendritic cells (DCs) or macrophages. The subsets of CD4^+^ Th cells can be classified according to the extracellular marker proteins, transcription factors, nuclear receptors, and cytokines that they produce [51]. CD4^+^ Th cells are activated by the interaction between APC and naïve CD4^+^ Th cells, a process comprising a major histocompatibility complex (MHC) class II molecule on an APC, which interacts with a T cell receptor on a naïve CD4^+^ Th cell. The MHC in humans is referred to as human leukocyte antigen (HLA), and HLA class II molecules consist of HLA-DP, HLA-DQ, and HLA-DR [52]. Naïve CD4^+^ Th cells can be differentiated into various CD4^+^ Th cell subsets via their interaction with APCs [53,54,55]. However, in addition to antigen presentation and recognition, T cell activation also requires co-stimulatory signaling. These co-stimulatory signaling pathways, also known as immune checkpoint pathways, are essential for the interactions between APCs and T cells, which subsequently facilitate the activation, proliferation, and differentiation of a naïve CD4^+^ Th cell. CD40, CD80, and CD86 are co-stimulatory molecules that provide co-stimulatory signals between APCs and CD4^+^ Th cells. CD40, also known as TNF Receptor Superfamily Member 5, is expressed on APCs, and its ligand is CD40 ligand (CD40L), also known as CD154, TNF ligand superfamily member 5, or TNF-related activation protein, is expressed on CD4^+^ Th cells. In addition, CD80, also known as B7-1, and CD86, also known as B7-2, are members of the B7 family of checkpoint proteins expressed on APCs, and their specific ligand is CD28 [56,57]. In addition, specific cytokines, which are secreted by DCs and other cells, induce stable expression of transcription factors and nuclear receptors (master regulators) that determine the differentiation of naïve T cells into specific Th subsets. CD4^+^ Th cell subsets include T helper 1 (Th1) [58], Th2 [58], Th9 [59,60], Th17 [61], Th22 [62], follicular helper T (Tfh) [63,64], and T-regulatory (Treg) cells [65,66]. LCA, DCA, and their derivatives have been shown to play crucial roles in influencing APCs (DCs and macrophages) (Table 1) and CD4^+^ Th cell subsets (Th1, Th17, and Treg cells) (Table 2). Essentially, APCs, Th1 cells, and Th17 cells promote immune responses, while Treg suppresses their responses [67].

### 3.2. Role of LCA, DCA, and Their Derivatives on DCs

**Figure 4:**
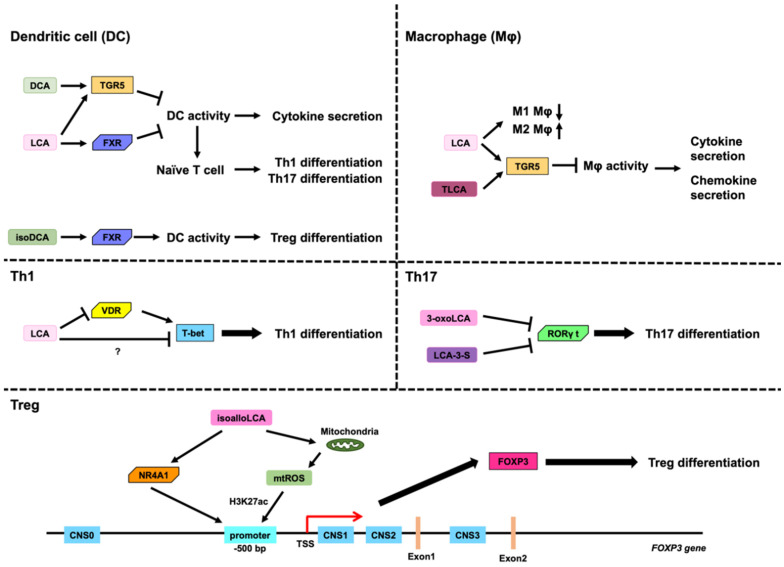
Role of LCA, DCA, and their derivatives on immune cells. Dendritic cells (DCs): DCA inhibits DC activity via TGR5, which in turn impedes the expression of proinflammatory cytokines, such as IL-1β, IL-6, IL-12, and TNF-α, and differentiation of naïve CD4^+^ Th cells into Th1 and Th17. LCA inhibits the expression of proinflammatory cytokines via TGR5 and FXR, and LCA-treated DCs reduce the differentiation of naïve CD4^+^ Th cells into Th1 or Th17 cells. In contrast, isoDCA induces Treg differentiation in the presence of DCs via FXRs in DCs. Macrophages (Mφ): LCA and taurine-conjugated LCA (TLCA) inhibit the expression of proinflammatory cytokines via TGR5. TLCA also inhibits the expression of chemokines, such as CCL2, CCL 3, CCL4, CCL5, CXCL9, and CXCL10 from macrophages via TGR5. In addition, LCA decreases the expression of hepatic M1 macrophages, which are responsible for the manifestation of proinflammatory effects, and increases hepatic M2 macrophages, which are responsible for anti-inflammatory effects. Th1 cells: LCA inhibits the differentiation of Jurkat cells into Th1 cells by inhibiting T-bet expression. In addition to VDR, this inhibition may involve other receptors (?) as well. Th17 cells: 3-oxoLCA and LCA 3-sulfate (LCA-3-S) inhibit the transcription activity of RORγt and Th17 differentiation with reduced IL-17a production. Treg cells: Human *FOXP3* and mouse *Foxp3* gene promoter regions are located in region of approximately 500 base pairs (bp) upstream of the transcription start site (TSS). In addition to the *FOXP3* promoter region, the conserved noncoding sequences (CNSs), CNS0, CNS1, CNS2, and CNS3, contribute to the promotion of *FOXP3* gene expression. IsoalloLCA increases mitochondrial reactive oxygen species (mtROS) levels, which in turn increases acetylated histone 3 lysine 27 (H3K27Ac) in the promoter region of the *FoxP3* gene, leading to the induction of the *FoxP3* gene. In addition, the increase in H3K27Ac levels in the promoter region by isoalloLCA is dependent on the presence of CNS3. IsoalloLCA also induces Foxp3 expression and Treg differentiation via NR4A1, and NR4A1 binds to 00 bp upstream of the *Foxp3* transcriptional start site.

Feeding diets containing DCA and LCA have been shown to reduce Th1 cells, Th17 cells, and CD11c^+^ MHC class II ^high^ DCs in the spleen of experimental autoimmune uveitis model mice. Moreover, DCA has been found to inhibit the secretion of proinflammatory cytokines, such as IL-1β, IL-6, IL-12, IL-23, and TNF-α, from lipopolysaccharide (LPS)-activated DCs [28]. DCA also inhibits the expression of CD40, CD80, CD86, and MHC class II in mouse DCs, but also the expression of CD40, CD86, and HLA-DR, which is a HLA class II molecule, in human DCs [28]. Although LPS-activated DCs induce the differentiation of naïve CD4^+^ T cells into Th1s or Th17s, DCA treatment impedes Th1 and Th17 differentiation [27]. In addition, DCA induces Takeda G protein receptor 5 (TGR5), also known as G protein-coupled bile acid receptor 1 (GPBAR1) or membrane-type receptor for bile acids (M-BAR), in LPS-activated DCs and inhibits the activation of nuclear factor-κB (NF-κB) via activation of TGR5 [27]. Thus, suppression of NF-κB activity via TGR5 activation by DCA may inhibit the expression of proinflammatory cytokines, such as IL-1β, IL-6, IL-12, and TNF-α, and cell surface markers, including CD40, CD80, CD86, MHC class II, and HLA-DR, in DCs (Figure 4) [27]. Moreover, LCA induces TGR5 and farnesoid X receptor (FXR), also known as NR1H4 [68], in DCs and inhibits the expression of proinflammatory cytokines, such as IL-1β, IL-6, IL-12, IL-23, and TNF-α, cell surface markers, including CD40, CD80, CD86, and MHC class II, via TGR5 in DCs. In addition, LCA-treated DCs impede the differentiation of naïve CD4^+^ Th cells into Th1 or Th17. In contrast, LCA inhibits the expression of proinflammatory cytokines via FXR. However, TGR5-mediated suppression of inflammatory cytokine expression by LCA is more potent than FXR-mediated suppression of inflammatory cytokine expression by LCA (Figure 4) [28].

IsoDCA leads to CD4^+^ T cell differentiation into Tregs in the presence of DCs, which, although dependent on FXR in the DCs, does not depend on naïve CD4^+^ Th cells. Despite the fact that the detailed mechanism by which isoDCA induces Treg differentiation is unknown, it is believed that isoDCA induces Treg cells by repressing genes whose expression is regulated by FXR in DCs (Figure 4) [24].

### 3.3. Role of LCA, DCA, and Their Derivatives on Macropahges

LCA and DCA both inhibit the secretion of TNFα from human macrophages, which are differentiated from human monocytes by the macrophage colony-stimulating factor and IFN-γ [69]. Furthermore, LCA significantly inhibits IL-1β secretion from mouse macrophages activated by nigericin, which induces IL-1β and IL-18 secretion via the Nod-Like Receptor family pyrin domain containing 3 (NLRP3) inflammasome [70,71] and LPS. The inhibition of IL-1β secretion by LCA and taurine-conjugated LCA (TLCA) is mediated by TGR5 (Figure 4) [25]. In contrast, DCA and taurine-conjugated DCA (TDCA) induce IL-1β secretion from mouse and human macrophages activated only by LPS [72]. TLCA also inhibits the expression of cytokines, such as IL-12 and TNFα, from LPS-activated human macrophages and also the expression of chemokines, CCL2, CCL 3, CCL4, CCL5, CXCL9, and CXCL10 from LPS-activated human macrophages. Downregulation of these chemokines by TLCA is regulated via the PKA pathway, which is likely activated by TGR5, but not the EPAC pathway (Figure 4). In addition, TLCA inhibits the migration of human NK cells to LPS-activated human macrophages [73]. 

Oral administration of LCA in mice changes the population of hepatic Kupffer cells (KCs), which are intraperitoneally injected with carbon tetrachloride (CCl_4_). Furthermore, LCA decreases hepatic M1 macrophages and increases hepatic M2 macrophages, which are responsible for the development of proinflammatory and anti-inflammatory effects, respectively (Figure 4) [74].

### 3.4. Role of LCA and DCA on Th1 Cells

Th1 cells mainly secrete interleukin-2 (IL-2), tumor necrosis factor α (TNF-α), and interferon (IFN)-γ, and they are involved in cellular immunity and the elimination of intracellular parasites. T-bet, also known as T-box transcription factor 21, signal transducer and activator of transcription 1 (STAT1), and STAT4 are involved in the differentiation of naïve CD4^+^ Th cells into Th1 cells and sustention to be Th1 cells. T-bet is considered a master regulator of Th1 cells [75].

LCA inhibits the expression of proinflammatory cytokines, such as IFNγ and TNFα, in Jurkat cells (human immortalized T lymphocyte cells) activated by phorbol myristate acetate (PMA)/ionomycin, mouse naïve CD4^+^ Th cells activated by PMA/ionomycin, and human naïve CD4^+^ Th cells activated by the CD3/CD28/CD137-coated Dynabeads T-activator [76]. LCA also impedes the differentiation of Jurkat cells into Th1 cells by inhibiting STAT1 and T-bet expression. These transcription factors are responsible for the differentiation of CD4 Th cells into Th1 cells and for IFNγ induction [77,78]. Jurkat cells express certain receptors for BAs, namely S1PR2, PXR, and VDR. VDR knockdown by small interfering RNA (siRNA) results in partial rescue from IFNγ and T-bet inhibition in Jurkat cells activated by PMA/ionomycin. S1PR2 activates kinases, including extracellular signal-regulated kinase 1 (ERK1) and ERK2, by phosphorylation [30]. However, LCA inhibits the phosphorylation of both ERK1 and ERK2 [76]. Thus, the inhibition of differentiation into Th1 cells by LCA may involve other receptors along with VDR (Figure 4). In addition, LCA and DCA decrease the spleen weight of mice and also decrease the number of Th1 and Th17 cells in the spleen [27].

### 3.5. Role of LCA, DCA, and Their Derivatives on Th17 Cells

Th17 cells mainly secrete IL-17A, IL-17F, and IL-22. Th17 cells play crucial roles in host defense against extracellular pathogens, particularly in mucosal and epithelial barriers. In contrast, abnormal activation of Th17 cells is associated with various autoimmune diseases, such as Crohn’s disease, rheumatoid arthritis, psoriatic arthritis, and multiple sclerosis [79]. Retinoid-related orphan receptor gamma t (RORγt) and STAT3 are involved in the differentiation of naïve CD4^+^ Th cells into Th17 cells and the sustention to be Th17 cells. RORγt is considered a master regulator of Th17 cells. The differentiation of Th17 cells in the intestine is mediated by gut bacteria and their metabolites, such as secondary BAs and SCFAs [80,81,82].

3-Oxo-LCA binds to RORγt, inhibiting its transcription activity. In addition, 3-oxo-LCA selectively inhibits Th17 differentiation with reduced IL-17a production. In contrast, the binding affinity of structurally similar BAs, 3-oxo-CA and 3-oxo-DCA, is approximately 20-fold weaker than that of 3-oxo-LCA. In addition, 3-oxo-CA and 3-oxo-DCA slightly inhibit differentiation into Th17 cells. Therefore, 3-oxo-LCA can inhibit Th17 differentiation by binding to and inhibiting RORγt (Figure 4). In contrast, 3-oxo-LCA has no effect on Th1 or Th2 differentiation [26]. It has been recently reported that the binding ability of LCA 3-sulfate (LCA-3-S) to RORγt is more potent than that of 3-oxo-LCA. Moreover, LCA-3-S inhibits Th17 differentiation via binding to RORγt (Figure 4). However, LCA-3-S has no effect on the differentiation of Th1, Th2, and Treg cells [83].

### 3.6. Role of LCA, DCA, and Their Derivatives on Treg Cells

Tregs mainly secrete suppressive cytokines, such as IL-10, IL-35, and transforming growth factor-β, and play important roles in suppressing immune responses as well as establishing and maintaining immune tolerance. There are three types of Tregs, namely thymus-derived Tregs (tTregs), peripherally derived Tregs (pTregs), and in vitro-induced Tregs (iTregs). Forkhead box P3 (FoxP3), which is a transcription factor and a master regulator of Tregs, and STAT5 are involved in the differentiation of naïve CD4^+^ Th cells into Tregs and the sustention to be Tregs. Tregs suppress immune responses by secreting suppressive cytokines, directly suppressing effector T cells, which are activated Th cells and CTLs, starvation of cytokines from T effector cells, such as IL-2, and modulating maturation and function of APCs [84]. The induction of differentiation to Tregs in the gut is essential for the establishment of immune tolerance to gut bacteria, a symbiotic relationship between host and gut bacteria, and maintaining the gut microbiota. In addition, the induction of Tregs in the gut is essential for facilitating tolerance against food antigens. 

IsoalloLCA selectively upregulates the expression of FoxP3 via the induction of *FoxP3* mRNA in mouse naïve CD4^+^ Th cells (Figure 4) [26,29]. However, other LCA derivatives, such as isoLCA and alloLCA, have not been found to enhance FoxP3 expression in mouse naïve CD4^+^ Th cells [26]. 3-oxo-alloLCA, which is an intermediate metabolite that produces isoalloLCA from CDCA, can weakly induce FoxP3 expression and differentiation into Treg cells [29]. Human *FOXP3* and mouse *Foxp3* gene promoter regions are located in a region of approximately 500 base pairs (bp) upstream of the transcription start site (TSS) [85,86]. In addition to the FOXP3 promoter region, the conserved noncoding sequences (CNSs), CNS0, CNS1, CNS2, and CNS3, contribute to the promotion of *FOXP3* gene expression. CNS0 is located upstream of the promoter region and functions as an inducer and stabilizer of *FOXP3* gene expression. CNS1 and CNS2 are located between TSS and exon 1 of the *FOXP3* gene, and they are responsible for *FOXP3* gene induction and stabilizing *FOXP3* gene expression, respectively. In contrast, CNS3 is located in the first intron of the *FOXP3* gene, and it is responsible for the induction of *FOXP3* gene expression [87,88,89,90,91,92]. Acetylated histone 3 lysine 27 (H3K27Ac) is an active epigenetic modification of histone H3, which has been found to increase in the promoter region, CNS1, CNS 2, and CNS 3 in Treg cells. The increase in H3K27Ac levels in the promoter region depends on the presence of CNS3 [93]. Although isoalloLCA treatment induces FoxP3 without any CNS1 and CNS 2 requirement, CNS3 is essential for FoxP3 induction by isoalloLCA [26]. Despite the fact that isoalloLCA treatment has no effect on cytoplasmic ROS levels, it induces the production of mitochondrial reactive oxygen species (mtROS), and differentiation into Treg cells by isoalloLCA is dependent on mtROS. In addition, mtROS induced by isoalloLCA are responsible for the increased levels of H3K27Ac in the *FoxP3* promoter region. Thus, isoalloLCA increases mtROS levels, which in turn increases H3K27Ac in the promoter region of the *FoxP3* gene, leading to the induction of the *FoxP3* gene (Figure 4). In addition, isoalloLCA has been shown to have no effect on the differentiation of naïve CD4^+^ Th cells into Th1 or Th2 cells [26]. The NGFI-B-responsive element (NBRE, AAAGGTCA, or TGACCTTT) is the nuclear receptor subfamily 4 group A (NR4A) family-binding site [94,95]. NBREs were identified by Assay for Transposase-Accessible Chromatin with high-throughput sequencing (ATAC-seq) when naïve CD4^+^ T cells were treated with isoalloLCA to induce Treg differentiation. The NR4A family consists of NR4A1, also known as NUR77 or nerve growth factor-induced-B (NGFI-B) [29], NR4A2, also known as NURR1 [96], and NR4A3, also known as neuron-derived orphan receptor 1 [97]. IsoalloLCA induces Foxp3 expression and Treg differentiation via NR4A1, as opposed to NR4A2 or NR4A3, and NR4A1 binds to 500 bp upstream of the *Foxp3* transcriptional start site, leading to the expression of Foxp3 (Figure 4) [29].

## 4. Concluding Remarks

LCA, DCA, and their derivatives are produced by the gut microbiota and function as signaling molecules that regulate immune cells, including DCs, macrophages, Th1 cells, Th17 cells, and Tregs, whose activities are directly associated with immune responses. The potential imbalance among immune cells is related to the development of various diseases, such as autoimmune and metabolic disorders [67,79]. Therefore, LCA, DCA, and their derivatives influence, either positively or negatively, the manifestation of diseases related to immune responses. Centenarians have unique gut bacteria that produce LCA derivatives, such as iso-, 3-oxo-, allo-, 3-oxo-allo-, and isoalloLCA. Thus, it is considered that longevity could be associated with BAs [48]. Senescent cells are a cause of age-related inflammation, and they are considered responsible for a variety of age-related diseases. Recent studies have shown that senescent cells disproportionally express programmed cell death ligand 1 (PD-L1), which is a ligand of the programmed cell death 1 (PD-1) immune checkpoint molecule, while senescent cells expressing PD-L1 accumulate with age. Senescent cells expressing PD-L1 are not eliminated because PD-L1 allows them to escape immune surveillance from CD8^+^ T cells [98]. PD-L1 expression is regulated by a variety of transcription factors and nuclear receptors, of which NF-κB is a major inducer of PD-L1 expression [56,99]. DCA inhibits the activation of NF-κB via triggering TGR5. In contrast, FXR activation increases PD-L1 expression [100]. Therefore, it is considered that BAs that have the ability to bind to TGR5 and/or FXR may not only regulate PD-L1 expression but also immune responses and senescence. Nuclear receptors and G protein-coupled receptors (GPCR) include orphan receptors whose endogenous ligands have not been identified yet [97,101]. BAs and their derivatives have a steroid structure, which is similar to the structure shown by various ligands for nuclear receptors. Moreover, BAs and their derivatives also act as ligands for GPCRs, such as TGR5. In addition, LCA, DCA, and their derivatives are ligands of orphan nuclear receptors, such as RORγt, NR4A1, and NR4A2 [97]. Therefore, it is essential to reevaluate the metabolites of BAs from the gut microbiota because they may act as ligands for various nuclear and cell surface receptors. Because microorganisms and their metabolites are closely related to human health and longevity, further investigations on microorganisms and their metabolism are highly expected. 

## Figures and Tables

**Table 1:** Bile acids and antigen-presenting cells.

Antigen-Presenting Cells	Bile Acids That Affect Antigen-Presenting Cells	Receptors for Bile Acids	Effects of Bile Acids
Dendritic cells	DCA, LCA	TGR5, FXR	Inductin of Th1 and Th17 differentiation
	isoDCA	FXR	Inductin of Treg differentiation
Macrophages	LCA, TLCA	TGR5	Cytokine and chemokine secretion

**Table 2:** Bile acids and helper T cell subsets.

T Cell Subsets	Differentiation Regulators	Characteristic Cytokines	Functions	Bile Acids That Affect T Cell Differentiation	Receptors for Bile Acids	Effects of Bile Acids
Th1	T-bet, STAT1, STAT4	IL-2, TNFα, IFNγ	cellular immunity, elimination of intracellular parasites	LCA	VDR	Th1 differentiation
Th17	RORγt, STAT3	IL-17A, IL-17F, IL-22	host defense against extracellular pathogens, barrier protection	3-oxoLCA, LCA-3-S	RORγt	Th17 differentiation
Treg	FoxP3, STAT5	IL-10, IL-35, TGF-β	suppressing immune responses, maintaining immune tolerance	isoallo LCA	NR4A1	Treg differentiation

## Data Availability

Not applicable.
